# Research progress of artificial intelligence in bone tumor imaging

**DOI:** 10.3389/fonc.2026.1780545

**Published:** 2026-03-11

**Authors:** Wenwei Zhang, Siwen Kang, Keda Li

**Affiliations:** 1 Liaoning University of Traditional Chinese Medicine, Shenyang, China; 2 Liaoning University of Traditional Chinese Medicine Affiliated Hospital, Shenyang, China

**Keywords:** artificial intelligence, bone tumors, deep learning, imaging, treatment assessment

## Abstract

This paper reviews the research progress of artificial intelligence (AI) in bone tumor imaging and explores its potential applications in improving diagnostic accuracy and clinical management. Bone tumors, including primary and metastatic tumors, often face the risk of misdiagnosis due to their rarity and diverse imaging characteristics, which significantly impacts patient prognosis. AI technologies, particularly deep learning (DL) algorithms, have been widely applied to the automatic recognition and segmentation of bone tumor regions in images, enhancing the efficiency and accuracy of radiological image analysis. Furthermore, AI plays a crucial role in the classification of bone tumors and the assessment of treatment efficacy, providing support for the development of individualized treatment plans. With the continuous advancement of AI technology, future research should focus on expanding its applications across different types of bone tumors and integrating multimodal imaging data to further strengthen clinical decision-making and patient management.

## 1 Introduction

Bone tumors refer to neoplastic lesions that occur in the bones and their associated tissues, such as blood vessels, nerves, and bone marrow. Clinically, they are classified into two main categories: primary and metastatic (1). Primary bone tumors originate from bone tissue itself. Although they account for only 0.2% of all cancers, they are the third leading cause of cancer-related death in individuals under 20 years of age, and their impact on adolescents is particularly profound (2, 3). Common types of primary bone tumors include osteosarcoma, Ewing sarcoma, and chondrosarcoma (4). These tumors are characterized by aggressive behavior and rapid progression, leading to significant bone destruction, pathological fractures, localized pain, and soft tissue swelling (2, 4). Additionally, primary bone tumors often impair motor function and can result in permanent disability in severe cases (5). Therefore, timely diagnosis and treatment are crucial to improving patient survival rates and quality of life. Imaging studies are the primary diagnostic tools for bone tumors, including X-radiation (X-ray), Computed Tomography (CT), Magnetic Resonance Imaging (MRI), and Positron Emission Tomography-CT (PET-CT) (6). These imaging techniques help determine the tumor’s exact location, size, boundaries, and the degree of bone destruction. They also assess whether the tumor has invaded surrounding soft tissues or metastasized to distant sites, providing essential information for clinical staging, prognosis evaluation, and treatment planning (7). However, because bone tumors are rare and exhibit highly diverse imaging characteristics, there is often significant overlap in the imaging presentations of different tumor types. This overlap poses a significant diagnostic challenge for radiologists, increasing the risk of misdiagnosis or missed diagnoses, which in turn affects patient outcomes (8). Therefore, enhancing radiologists’ sensitivity to the imaging features of bone tumors and developing more advanced imaging analysis techniques are critical to improving the accuracy of bone tumor diagnosis and ensuring optimal patient management.

AI was first introduced at the Dartmouth Conference in 1956 (9). Its core idea is to develop systems capable of performing specific tasks by simulating human intelligent behavior and cognitive processes using computer technology (10, 11). With advancements in medical imaging and computer technology, AI is increasingly used in medical imaging. AI employs DL and image recognition to help doctors analyze CT, MRI, and X-ray images more accurately, enabling the detection of early lesions and small abnormalities, thus improving early diagnosis accuracy (12). DL, a key technology in AI derived from artificial neural networks, constructs multi-layer neural networks that simulate human brain information processing, enabling automatic learning and feature extraction (13, 14). DL relies on efficient algorithms like Fully Convolutional Networks (FCN), Convolutional Neural Network (CNN), and Stacked Autoencoders (SAN), which are commonly used in medical image analysis for tasks such as organ and lesion segmentation, anomaly detection, and classification (15). Different DL architectures have unique technical characteristics that make them highly adaptable to the morphological and texture features of bone tumor imaging: CNN excels at extracting local spatial features of bone tissue and tumor lesions (16), FCN realizes end-to-end pixel-level segmentation suitable for accurate identification of tumor boundaries (17), and SAN is good at unsupervised learning of low-dimensional abstract features from raw bone tumor imaging data, and can effectively extract and reconstruct the intrinsic texture, gray-scale and density features of bone tissue and tumor foci while reducing data dimensionality and suppressing imaging noise (18). Currently, AI applies DL for the early detection and classification of bone tumors, automatically identifying tumor features in X-rays, CT scans, and MRIs, distinguishing them from normal tissue, and assessing their potential benign or malignant nature, thus aiding clinical treatment decisions.

This literature review adheres to the PRISMA (Preferred Reporting Items for Systematic Reviews and Meta-Analyses) guidelines to maintain transparency and thoroughness in the literature selection process. The research team performed searches in the PubMed and Web of Science databases for studies published between 2005 and 2025, utilizing keywords such as “bone tumors”, “artificial intelligence”, “medical imaging”, “deep learning”, “image segmentation” and “prognostic prediction.” Due to the uncommon nature of bone tumors and the swift advancements in AI technology, a quantitative meta-analysis was not conducted in this study. Instead, a two-phase literature screening approach was adopted: the first phase involved an initial review of titles and abstracts to identify significant studies and influential original research; the second phase utilized a “snowball” technique to explore references from key articles to ensure comprehensive coverage of related research. The authors meticulously assessed the data quality, model validation methods, and clinical implications of all selected studies to confirm the scientific integrity and reliability of the conclusions presented in this review.

## 2 The application of AI in the recognition and segmentation of bone tumor regions

MRI is essential for diagnosing bone tumors, offering high-resolution images that help doctors assess tumor shape and location (19). However, manual evaluation is time-consuming and subjective, impacting diagnostic accuracy. Thus, integrating AI models to aid in MRI image analysis is crucial. Dionísio et al. (20) evaluated the reproducibility of manual segmentation of MRI images using 20 pathologically confirmed cases of bone tumors and compared it with semi-automated segmentation. The results showed that the Dice Similarity Coefficient (DSC, a metric to measure the overlap degree of segmented regions and gold standard, with a value range of 0–1, and the closer to 1, the higher the segmentation accuracy) values for semi-automated segmentation ranged from 0.71 to 0.96, with Hausdorff Distance (HD, a metric to measure the maximum distance between the segmented boundary and the gold standard boundary, the smaller the value, the higher the matching degree of the segmentation boundary) values of 5.38 to 31.54 mm, and it significantly reduced segmentation time (*P ≤* 0.05), indicating that semi-automated segmentation is an effective and efficient option. Qu et al. (21) developed a DL-based automatic segmentation method for MRI and evaluated it on datasets from 90 and 15 patients, respectively. The results indicated that the method achieved an HD98 (the 98th percentile of HD, which reduces the impact of extreme values on the evaluation result) of 3.62 mm, a DSC of 0.85, and a success rate of 97.78% on the development dataset; on the test dataset, the HD98 was 3.92 mm, the DSC was 0.888, and the success rate was 93.33%. The segmentation time was significantly reduced from 1820.3 seconds for manual annotation to 19.2 seconds, resulting in a 100-fold speed increase. The multi-view CNN model adopted in this study effectively fuses the image features of different views of the pelvis, making up for the deficiency of single-view feature extraction and thus improving the segmentation accuracy of pelvic tumors with complex anatomical structures. These results demonstrate that the DL segmentation framework can quickly and accurately perform MRI segmentation of pelvic tumors, significantly optimizing the surgical planning process.

To enhance model performance, Wu et al. (22) proposed an AI multi-processing solution for osteosarcoma segmentation that combines pre-screening, denoising, and segmentation techniques. Their model, the Embedded Transformer layer within a U-Net (ETUNet), leverages U-Net’s local feature extraction and Transformer’s long-range dependency capture, making it particularly effective for tumors with irregular boundaries and diverse morphologies. They employed a Sliding Block Filter (SBF) to select valuable lesion images and an Optimized Non-Local Means (NLM) algorithm for denoising. Segmentation was performed using ETUNet combined with Conditional Random Fields (CRF). Experiments on over 70,000 MRI images from Chinese hospitals achieved a pre-screening accuracy of 95.67% when λ_T was set between 130 and 150. Final segmentation metrics showed a DSC of 0.935 and an Intersection over Union (IoU) of 0.919, surpassing traditional models by over 3% while maintaining low computational costs. Grad-CAM was used to visualize the ETUNet’s decision-making process, highlighting tumor regions and enhancing clinician trust in the AI results. This approach significantly improves the efficiency and accuracy of osteosarcoma diagnosis.

In summary, the integration of AI and MRI for bone tumor segmentation offers notable benefits, including enhanced diagnostic accuracy, efficiency, and consistency. However, current research is primarily focused on MRI, with limited exploration of other imaging techniques like CT and X-rays. Many models target specific tumor types, restricting broader applicability. Additionally, the lack of standardized data collection hinders interoperability among medical institutions, limiting clinical AI model applications. Future studies should explore various imaging methods and tumor types, establish standardized imaging datasets, and adopt interpretable AI approaches to enhance clinical acceptance and practicality.

## 3 Research and application of AI in bone tumor classification

The distinction between benign and malignant bone tumors is crucial for choosing the surgical treatment plan (23), and imaging examinations are key methods for this purpose. Eweje and his team (24) developed a DL algorithm to differentiate between benign and malignant bone lesions. This model was built on a retrospective multi-center dataset, which effectively improved the generalizability of the model compared with single-center models. Based on routine MRI and patient information, the study included 1,060 histologically confirmed bone lesions for validation. The results showed that the model’s performance in classification accuracy, sensitivity, and specificity was comparable to that of radiology experts, with values of 76%, 79%, and 75%, respectively, and a Receiver Operating Characteristic (ROC) curve area under the curve (AUC) of 0.82 for giant cell tumor of bone (GCTB). In external testing, the ROC AUC was 0.79. This study supports the development of AI-assisted diagnostic tools aimed at reducing unnecessary referrals and biopsies. He et al. (25) developed a DL model to assess its discriminatory ability for benign vs. non-benign and malignant vs. non-malignant classifications, while also evaluating its performance in a three-class classification (benign, intermediate, and malignant). The study validated the model’s generalizability on an external test set and compared its performance with the interpretations of five radiologists with varying levels of experience. The results showed that the model achieved AUCs of 0.894 and 0.877 for benign vs. non-benign classification, and AUCs of 0.907 and 0.916 for malignant vs. non-malignant classification. In the three-class classification, the model’s accuracy was 72.1%, and 73.4% in external testing, outperforming most junior radiologists.

Schacky et al. (26) studied a multitask DL model for bounding box placement, segmentation, and classification of primary bone tumors on radiographs. This multitask learning framework enables joint training of related tasks, allowing feature sharing that enhances overall model performance. It is particularly well-suited for clinical applications requiring simultaneous tumor localization, segmentation, and classification. The study analyzed radiographs from 934 patients, and the results indicated that the model achieved an accuracy of 80.2% in classifying benign and malignant bone tumors, with a sensitivity of 62.9% and specificity of 88.2%. The model’s accuracy was higher compared to that of two radiology residents. The study by Liu et al. aims to construct and validate a fusion model that combines DL and machine learning for the classification of benign, malignant, and intermediate bone tumors. The fusion model first uses DL models (VGG16, ResNet50) to extract deep features of images, and then uses traditional machine learning models (SVM, Random Forest) to classify the deep features, which makes up for the deficiency that a single DL model is prone to overfitting on small sample datasets. The research data consists of 982 images from 643 patients. The results indicate that in the binary classification task, the AUC of the fusion model was 0.898, 0.894, and 0.865, outperforming the individual radiological models. In the three-class classification task, the macro-average AUC of the fusion model was 0.872, which is higher than the 0.813 of the radiological models. Additionally, the average AUC of all radiologists was 0.819, which improved by 0.026 after using the fusion model. Overall, the classification performance of this model is comparable to that of senior radiologists, providing support for the differential diagnosis of bone tumors (27). In summary, although imaging AI models have demonstrated good diagnostic accuracy in classifying bone tumors, comparable to that of experienced radiologists, caution is still necessary in clinical application. These technologies should be used in conjunction with the judgment of medical professionals to ensure patient safety and treatment efficacy. Additionally, the diversity of training data and validation will impact the generalizability of the models, necessitating broader validation and assessment.

## 4 Research on the application of AI in the assessment of treatment efficacy and prognosis prediction of bone tumors

The traditional treatment for bone tumors primarily includes neoadjuvant chemotherapy (NAC) in combination with surgery and postoperative therapy, aimed at shrinking the tumor and improving surgical success rates (28, 29). However, poor NAC responses often lead to a poor prognosis, indicating a need for new treatment strategies. Current assessments of pathological response rely on postoperative samples, which have limitations. Radiological examinations serve as effective preoperative tools to predict responses to NAC and guide clinical decisions. Zhong et al. (30) studied how to predict the response of osteosarcoma patients to NAC using automatic segmentation technology and radiomics scoring. The study involved a total of 144 patients, divided into training and test groups. The nnU-Net model was used for automatic segmentation of the Region of Interest (ROI) in preoperative MRI, and radiomic features were extracted. The nnU-Net model has the advantage of adaptive network architecture and parameter adjustment, which is especially suitable for the segmentation of medical images with diverse anatomical structures, and its segmentation accuracy is higher than the traditional U-Net model. The results showed that 25% of the patients were good pathological responders (pGRs), with a Dice coefficient of 0.869 for the segmentation model. The AUCs for the clinical model and the radiomics model were 0.636 and 0.759, respectively, while the AUC for the clinical-radiomics nomogram was 0.793, with an accuracy of 79.1%. The nomogram proposed in this study could assist radiologists in identifying patients who have a good response to NAC. Huang et al. (31) conducted a study utilizing multi-parameter MRI combined with machine learning to assess tumor necrosis in patients with osteosarcoma after NAC. The study included 12 patients and 102 tissue samples. The results showed a significant improvement in differentiation capability when combining multi-parameter MRI: the AUC for distinguishing survival from non-survival in non-chondroid tumors increased from 0.93 to 0.97; the AUC for distinguishing survival from non-survival in tumors increased from 0.83 to 0.90; and the AUC for distinguishing survival from non-survival in chondroid tumors increased from 0.61 to 0.81. The fusion of multi-parameter MRI features can comprehensively reflect the biological characteristics of tumors, which is far better than single-parameter MRI in evaluating tumor necrosis after chemotherapy. This method also provides an objective and accurate tool for evaluating the response of osteosarcoma to NAC.

A study aimed to predict early postoperative recurrence of spinal GCTB through radiomics analysis based on preoperative CT. A total of 62 patients were included in the study, with an average follow-up of 73.7 months. The recurrence rate was found to be 27.4%, and patients who underwent curettage had a significantly higher recurrence rate compared to other surgical methods. The final radiomics model utilized 10 features, achieving an accuracy of 89% and an AUC of 0.78 (32). He et al. (33) utilized a deep CNN (DCNN) to predict local recurrence of GCTB after surgical treatment, analyzing MRI images from 56 patients and assessing accuracy using four-fold cross-validation. The CNN achieved an accuracy of 75.5%, while a logistic regression model that included patient age and tumor location improved accuracy to 78.6%, both surpassing the 64.3% accuracy of radiologists. In terms of sensitivity, the CNN and regression model achieved 85.7% and 87.5%, respectively, significantly outperforming the radiologists’ sensitivity of 58.3%. This study suggests that CNNs have potential in predicting GCTB recurrence, and integrating clinical characteristics can further enhance predictive accuracy. Integrating clinical characteristics with imaging features has become an important development trend of AI-based tumor prognosis prediction models.

In conclusion, AI technology shows significant efficiency and accuracy in assessing bone tumors. Unlike traditional pathological response assessments that rely on postoperative samples, AI evaluations based on imaging can be performed preoperatively, offering earlier guidance for clinical decision-making and optimizing personalized treatment plans. However, current studies have limitations. First, the generally small sample sizes restrict the broader applicability of the results; for instance, Zhong’s study included only 144 patients, while Huang’s had just 12. Second, although AI models outperform traditional methods in accuracy, their reliance on high-quality and standardized imaging data limits their applicability across different hospitals or devices. Additionally, the “black box” nature of AI models may reduce clinicians’ trust in their predictions, highlighting the need for clear explanations of the model’s decision-making processes for clinical use.

## 5 Analysis of the application potential of AI in metastatic bone tumors

Metastatic bone tumors are cancer cells that spread to the bones from primary tumors, often occurring in the late stages of cancers like breast, lung, and prostate cancer (34). These tumors can be classified as osteolytic or osteoblastic. Osteolytic metastases, common in breast and lung cancers, cause bone tissue destruction, while osteoblastic metastases, primarily linked to prostate cancer, promote bone formation and increase density (35). CT scans can detect metastatic bone tumors, but the process is complex and time-consuming, leading to missed diagnoses due to the small size of some lesions and overlapping features with normal bone tissue. AI-assisted detection technologies may reduce these missed diagnoses and improve accuracy. Huo et al. (36) studied aDCNN model for the automatic detection of bone metastases from lung cancer in CT scans. Based on CT data from 126 patients, the results showed that the model achieved a detection sensitivity of 89.4% in the testing cohort, with an average of 5.24 false positives per case and a Dice coefficient of 0.856 for segmentation. In collaboration with radiologists, the detection accuracy of three junior radiologists improved from 61.7% to 87.9%, and their sensitivity increased from 68.0% to 90.2%, while the average interpretation time per case was reduced by 228 seconds. The DCNN model adopted in this study uses the anchor-free detection framework, which is more suitable for the detection of metastatic bone lesions with small size and irregular shape, and effectively reduces the missed diagnosis rate of small metastatic lesions. Lin et al. (37) studied the use of DL technology for the automated diagnosis of bone metastasis in single-photon emission CT (SPECT) nuclear medicine imaging. They developed several deep network-based classifiers focused on the classification of SPECT bone images. The specific steps included cropping the thoracic region of the SPECT images, performing data augmentation, and constructing and fine-tuning deep classifiers based on VGG, ResNet, and DenseNet. The results indicated that the proposed classifiers performed exceptionally well in identifying bone metastasis, achieving an accuracy of 0.9807, precision of 0.9900, recall of 0.9830, specificity of 0.9890, F1 score of 0.9802, and AUC of 0.9933. This research provides an effective solution for the automated diagnosis of bone metastasis in SPECT imaging. The above research indicates that AI has significant potential in the detection and diagnosis of metastatic bone tumors. However, the studies have limited sample sizes and primarily focus on specific cancer types and imaging modalities, so the generalizability of their results still requires further validation. In addition, current AI models only focus on the qualitative detection of metastatic bone tumors, and the research on the quantitative assessment of the severity of bone metastasis and the prediction of treatment response is relatively insufficient, which is an important direction for future research.

## 6 Summary and outlook

In summary, the application of AI in bone tumor imaging has significantly improved the accuracy of early detection, optimized lesion segmentation, and shortened diagnosis times. Through DL algorithms, AI can efficiently analyze imaging data to assist doctors in making precise assessments, thereby reducing errors. However, current research also has some limitations. Firstly, most studies lack multi-center prospective trials, resulting in generally small sample sizes; many clinical trials involve only dozens to a few hundred cases. This limited sample data makes it difficult to support the widespread clinical validation of models, affecting their generalizability. Secondly, existing automatic segmentation models and specific diagnostic classification models are not sufficiently diverse, often targeting only specific types of bone tumors, while their ability to identify other tumor types is relatively inadequate. Additionally, most studies rely on single-modal imaging data and fail to fully utilize the advantages of multimodal imaging, which limits the overall practicality of the models. Furthermore, the integration of clinical information is often minimal, leading to relatively limited assistance from models in complex clinical scenarios.

With the continuous advancement of AI and medical imaging technology, the application of AI in bone tumor imaging is progressing into a new development stage. Future research should focus on several directions to promote the clinical translation of AI technology in the diagnosis and treatment of bone tumors. Firstly, there is a need to establish standardized multi-center imaging datasets for bone tumors through collaboration among multiple institutions and data sharing to address the problem of insufficient sample sizes. This includes systematically collecting prospective multi-center bone tumor imaging data along with corresponding clinical information, laboratory indicators, and follow-up data. Additionally, synthetic data strategies (such as GANs and diffusion models) can be employed to supplement real data, thereby reducing the risk of model overfitting. Secondly, future AI models should integrate multimodal imaging data (such as X-rays, CT scans, MRI, etc.) and multidimensional clinical information to comprehensively reflect the characteristics of bone tumors, improving diagnostic and prognostic accuracy. Furthermore, incorporating Traditional Chinese Medicine diagnostic information into AI models can explore the organic combination of Eastern and Western medicine in the diagnosis and treatment of bone tumors, providing new ideas for personalized treatment.

In terms of algorithm innovation, attention should be paid to the fusion of multiple technologies and the application of cutting-edge methods. On one hand, it is essential to strengthen the combination of interpretable handcrafted features from radiomics with high-dimensional deep features from DL, balancing model accuracy and interpretability. On the other hand, fine-tuning large pre-trained medical imaging models on small sample bone tumor datasets can utilize federated learning technology to address data privacy and silos. Additionally, advancing the study of imaging-genomics relationships can reveal the molecular mechanisms behind imaging phenotypes, providing scientific evidence for precise treatment of bone tumors. Addressing the “black box” problem of AI models is key to enhancing clinical acceptance. Future efforts should explore the application of explainable AI methods such as Grad-CAM, SHAP, and LIME in bone tumor imaging models to visualize the decision-making process and feature contributions of the models. Large-scale clinical validation is also necessary to assess the impact of explainable AI on the efficiency and accuracy of clinical diagnoses, thereby strengthening clinicians’ trust in AI models.

The clinical translation and industrialization process of AI requires close collaboration among researchers, clinicians, and regulatory agencies such as the National Medical Products Administration (NMPA) and the U.S. Food and Drug Administration (FDA) to jointly formulate technical standards and clinical application guidelines for bone tumor imaging AI models. By seamlessly integrating AI models with hospital PACS/RIS/HIS systems, we can minimize disruptions to clinical workflows. Additionally, developing lightweight algorithms and edge computing technologies will promote the deployment of AI models in primary healthcare institutions, mobile terminals, and telemedicine, expanding the coverage of intelligent bone tumor diagnostic technology.

In conclusion, AI holds immense application potential and development prospects in the field of bone tumor imaging. Through collaborative efforts across multiple centers, standardized data construction, algorithmic innovations, and rigorous clinical validation, AI will play a crucial role in the early diagnosis, precise classification, efficacy evaluation, and prognostic prediction of bone tumors, driving the evolution of bone tumor diagnosis and treatment towards intelligence and precision, ultimately significantly improving patient survival rates and quality of life.
